# Rare synchronous caecal and sigmoid volvulus: management of two cases

**DOI:** 10.1093/jscr/rjaa556

**Published:** 2021-01-29

**Authors:** Butt Muhammad, Lee Alice, Aseem Rabiya, Smith Jason

**Affiliations:** West Middlesex University Hospital, General Surgery, Isleworth, UK; West Middlesex University Hospital, General Surgery, Isleworth, UK; Chelsea and Westminster Hospital NHS Foundation Trust, General Surgery, London, UK; West Middlesex University Hospital, Colorectal surgery, Isleworth, UK

## Abstract

Colonic volvulus is one of leading causes of large bowel obstruction following colorectal cancer and diverticulitis, accounting for 5% of cases. Sigmoid volvulus is most common (75%) followed by volvulus of the caecum (15%), transverse colon (3%) and splenic flexure (2%). Synchronous volvulus of the caecum and sigmoid is very rare, with six reported cases in the literature to the best of our knowledge. We report two cases within 6 months. The key learning points include that classical radiological signs of both caecal and sigmoid volvulus may not be present, and that prompt, definitive management is necessary to prevent recurrence and morbidity.

## BACKGROUND

Synchronous caecal and sigmoid volvulus is extremely rare, with six reported cases in the literature to our knowledge. We chose to highlight the following two cases to practising surgeons given the rarity of the presentation, the difficulty in radiological diagnosis and the associated morbidity without prompt, definitive management.

## CASE 1

### Presentation

A 73 years old male with a previous history of myocardial infarction, hypertension and hypercholesterolemia presented to our emergency department with generalized abdominal pain, distension and constipation for 1 week. He had multiple previous attendances with the same presentation (five admissions in 8 months), previously diagnosed as sigmoid volvulus and managed with either flatus tube insertion and/or flexible sigmoidoscopy. In all cases, decompression was initially successful with resolution of symptoms and signs of large bowel obstruction. He was discharged home with planned follow-up in the colorectal clinic including discussion for elective surgery to definitively manage the recurrent sigmoid volvulus. Unfortunately, he re-presented before outpatient work-up for surgery was possible. On re-admission, his examination revealed a distended, tender abdomen with stable observations and no evidence of peritonism.

### Investigations

**Figure 1:**
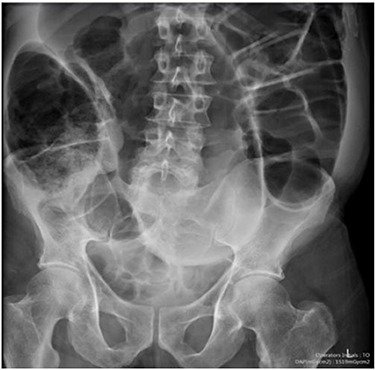
Case one: plain abdominal film showing dilated large bowel and the ‘coffee bean’ sign associated with sigmoid volvulus.

**Figure 2:**
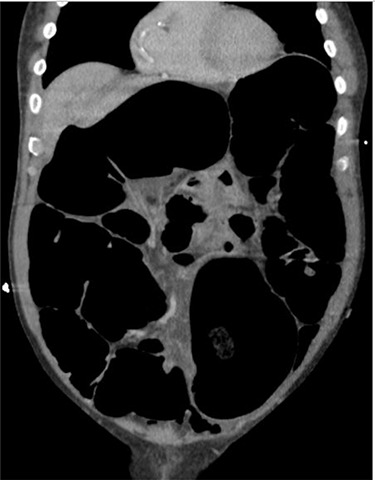
Case one: coronal slice of a CT scan of the abdomen and pelvis with intravenous contrast showing dilated large bowel.

Blood tests on admission were unremarkable. Each admission, the patient’s plain abdominal films showed dilated large bowel loops consistent with sigmoid volvulus (Fig. 1). A subsequent computed tomography (CT) of the abdomen and pelvis with intravenous contrast revealed dilated large bowel and was reported as sigmoid volvulus with no evidence of perforation (Fig. 2). Caecal volvulus was not appreciated on this scan.

### Management

On the patient’s sixth admission, the decision was taken for definitive surgery in the form of a subtotal colectomy and ileostomy as he was not responding to conservative treatment despite endoscopic decompression. Intraoperative findings revealed a grossly distended sigmoid and right colon from a 360 degree twist. Mesentery at the recto-sigmoid junction was ischaemic with an ischaemic patch on rectum. Histological findings included congestion, oedema and haemorrhage consistent with the effects of volvulus.

### Follow-up

**Figure 3:**
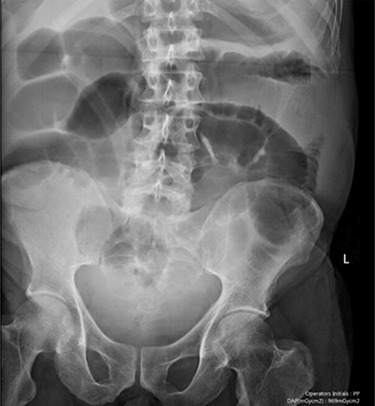
Case one: a plain abdominal film consistent with postoperative ileus.

The patient recovered well and was discharged on the sixth postoperative day. Unfortunately, he re-presented on day 11 post-operatively with vomiting. A plain abdominal film was consistent with ileus (Fig. 3), which was managed with wide bore nasogastric tube insertion, fluid and electrolyte balance. He was discharged and reviewed in clinic approximately 2 months later, with complete resolution of symptoms and a healthy stoma. He has since been discharged from regular follow-up.

## CASE 2

### Presentation

A 79 years old male with a history of hypercholesterolemia, hypertension and benign prostatic hypertrophy presented with generalized abdominal pain, constipation and distension of 4 days duration. He had presented twice previously (within the previous month) with the same symptoms. As in case one, he was initially diagnosed as having sigmoid volvulus and decompressed using flexible sigmoidoscopy. An outpatient CT pneumocolon post-decompression and did not show any further evidence of volvulus, colonic polyps or cancer. On this admission, he had a distended, tender abdomen with no evidence of peritonism and stable observations.

### Investigations

**Figure 4:**
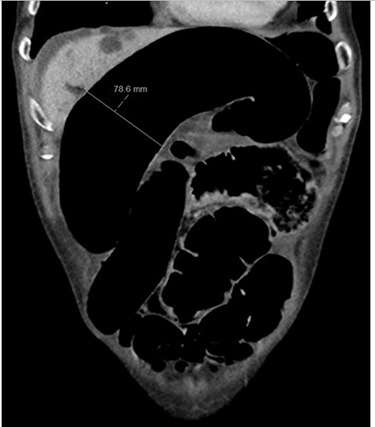
Case two: a coronal slice of a CT scan of the abdomen and pelvis with intravenous contrast showing dilated large bowel loops; the radiology report suggested sigmoid volvulus with a centrally lying caecum.

**Figure 5:**
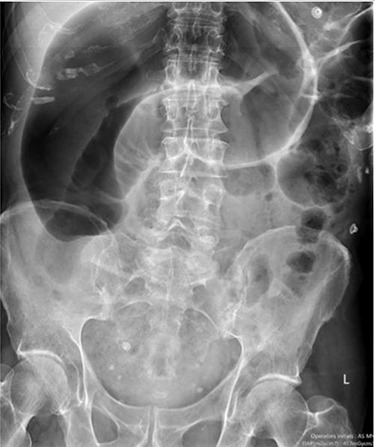
Case two: a plain abdominal film showing dilated large bowel and the ‘inverted comma’ sign associated with caecal volvulus.

Blood tests on admission were unremarkable, with normal electrolytes levels and lactate. A plain abdominal film showed a typical appearance of caecal volvulus, with an inverted ‘comma sign’ (Fig. 4). A CT scan of the abdomen and pelvis with intravenous contrast was reported as sigmoid volvulus with the involved loops sitting predominantly in the right upper quadrant immediately inferior to the liver (Fig. 5).

### Management

**Figure 6:**
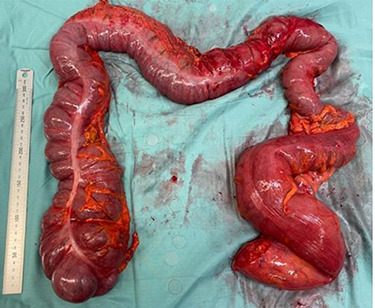
Case two: subtotal colectomy specimen showing dilated caecum and sigmoid colon.

As the patient did not demonstrate evidence of peritonism or ischaemia, conservative management was trialled overnight. Whilst the patient’s pain initially improved, the subsequent day he was still unable to tolerate oral intake and a repeated abdominal film showed persistent volvulus. He was taken for an exploratory laparotomy, which showed grossly distended caecum on a hypermobile mesentery with classic anticlockwise twist (Fig. 6). A simultaneous sigmoid volvulus was identified extending up the right paracolic gutter into the epigastric region, as the caecum had rotated upward creating space for the sigmoid to sit on the right-hand side. As the entire colon except the splenic flexure was grossly distended and hypermobile, the decision was taken to do a subtotal colectomy and end ileostomy (the rectum was considered too dilated for a primary anastomosis).

### Follow-up

On the fourth postoperative day the patient developed generalized abdominal tenderness and hiccups. A CT scan of the abdomen and pelvis performed on the same day was consistent with paralytic ileus. The patient continued to have poor oral intake with nausea. A second CT scan of the abdomen and pelvis was therefore performed on the 15th postoperative day. This showed dilated small bowel loops with a possible transition at the site of the ileostomy. He was managed conservatively with wide bore nasogastric tube and Foley catheter insertion into the ileostomy. He fully recovered and was discharged 4 weeks post-operatively. He has since been reviewed in colorectal clinic 4 months post-operatively. He reported full return to normal activities and diet and the stoma remains healthy.

## DISCUSSION

Current recommendations for management of sigmoid volvulus depend on clinical status [2]. In the reported cases, non-surgical decompression was trialled for presumed sigmoid volvulus. Roy *et al*. [3] also attempted conservative management in a stable patient with flexible sigmoidoscopy and rectal tube insertion. This was ultimately unsuccessful with recurrence following removal of the rectal tube. This suggests that non-surgical management in patients with simultaneous caecal and sigmoid volvulus is not a satisfactory long-term treatment option. The patient described in case one was discharged with a view to work-up for elective surgery, however he unfortunately re-presented in large bowel obstruction before this was possible.

Given the synchronous volvuli in these cases, a subtotal colectomy with end ileostomy was performed with an option for later reversal. The decision for ileostomy was based on the presence of multiple risk factors for anastomotic leak in these patients, including age, gender, comorbidities and intraoperative findings [4]. Definitive surgical management described in previous case reports was usually subtotal colectomy with either end ileostomy [3, 5] or ileorectal anastomosis [6, 7]. Singh *et al*. [8] reduced both volvuli, performed caecostomy and fixed part of the sigmoid colon to the transverse colon to reduce recurrence. The decision to form an ileostomy versus ileorectal anastomosis should be decided by the surgical team on a case-by-case basis, taking into account known risk factors for anastomotic breakdown [4].

## CONCLUSIONS

As take-home messages, we recommend that surgeons consider synchronous large bowel volvulus as a rare and easily missed cause of large bowel obstruction, particularly with recurrent presentations of conservatively managed sigmoid volvulus. Radiological diagnosis is challenging, often suggesting only one type of large bowel volvulus and definitive diagnosis is made intra-operatively.

## CONFLICT OF INTEREST STATEMENT

None declared.
